# Electric Field Induced Dewetting of Hydrophobic Nanocavities at Ambient Temperature

**DOI:** 10.3390/nano10040736

**Published:** 2020-04-12

**Authors:** Chenchao Li, Dongdong Lin, Wenhui Zhao

**Affiliations:** School of Physical Science and Technology, Ningbo University, Ningbo 315211, China; 1711071045@nbu.edu.cn

**Keywords:** dewetting, electric field, hydrophobic nanocavities, molecular dynamics simulation

## Abstract

The understanding of water dewetting in nanoporous materials is of great importance in various fields of science and technology. Herein, we report molecular dynamics simulation results of dewetting of water droplet in hydrophobic nanocavities between graphene walls under the influence of electric field. At ambient temperature, the rate of dewetting induced by electric field is significantly large. Whereas, it is a very low rate of dewetting induced by high temperature (423 K) due to the strong interaction of the hydrogen-bonding networks of water droplets in nanocavities. In addition, the electric filed induced formation of a water column has been found in a vacuum chamber. When the electric field is turned off, the water column will transform into a water droplet. Importantly, the results demonstrate that the rate of electric field-induced dewetting increases with growth of the electric field. Overall, our results suggest that electric field may have a great potential application for nanomaterial dewetting.

## 1. Introduction

In spite of the hydrophobic nature of nonpolar nanocavities, however, water can be adsorbed inside these hydrophobic nanopores without the application of a high-pressure [1,2,3,4]. So far, a series of experimental and theoretical studies have demonstrated that nanocavities in single-wall carbon nanotubes (SWNTs) and graphene walls can encapsulate water molecules [5,6,7,8,9,10]. Many studies on water in these nonpolar nanocavities have mainly focused on the structures and thermodynamic properties which are fundamentally different from bulk water [11,12,13,14,15]. For example, water confined nanopores can form unique ordered ice and exhibit anomalous diffusion or transport behavior [10,11,12,13,14,15,16,17]. On the other hand, the understanding of water dewetting in nanoporous materials is also significant in various fields including nanomaterial drying, oil recovery, soil remediation, energy conversion, storage applications, and protein folding [18,19].

The dewetting transition phenomenon in-between hydrophobic nanocavities immersed in aqueous solutions has been investigated extensively [20,21,22,23,24]. Huang et al. reported the observation of spontaneous dewetting when the separations of two walls are smaller than the critical distance [21]. Amabili et al. found that pore morphology can determine spontaneous liquid extrusion from nanopores [24]. The dewetting transition can be enhanced by the aggregated hydrophobic and hydrophilic molecules [25,26,27,28,29]. Li et al. reported that the nitrogen molecules aggregate in the vicinity of the two hydrophobic plates and exclude water molecules when hydrophobic plates immersed in nitrogen aqueous solutions [25]. In addition, alcohols, trimethylamine-n-oxide (TMAO), and urea are also found to promote dewetting transition in-between hydrophobic nanocavities immersed in aqueous solutions [26,27,28,29].

Another strategy of dewetting is by applying the electric fields. It is well known that electric field can dramatically change the phase behavior of water [30,31,32,33,34,35,36,37,38]. Electric fields always promote the freezing of both bulk and confined water through electric-field-induced realignment of the dipoles of water molecules [30,31,32,33,34]. And the electromelting of monolayer ice was found by applying the vertical electric field [35]. It is also reported that the electric field can induce the evaporation of water confined in-between hydrophobic nanocavities immersed in aqueous solutions [37]. Furthermore, wetting and dewetting of narrow hydrophobic nanocavities induced by electric fields have been studied by Kayal and Chandra [39]. These studies have mainly focused on the system of either end of the nanopores in contact with a liquid water bath. On the other hand, the formation of water droplets (or cluster) was observed when water molecules trapped in nanocavities exposed to water vapor or to atmosphere [40,41,42]. It would be hard for the water droplets to be excluded because the energy barrier of water removed from the nanocavities is higher than that of water filling the nanocavities [43]. 

Although thermal drying is widely used to remove the capillary water, high energy consumption limits the application of thermal drying. On the contrary, electric field-assisted dewatering can accelerate the sludge dewatering process with low energy consumption (~0.12–0.5 kWh/kg_water_) compared with thermal drying (~0.61–1.2 kWh/kg_water_) [44,45]. Wan et al. demonstrated that the fast water harvesting and drying are induced by negative and positive bias using N-doped graphene micropads [46]. Hens et al. found that the vertical electric field enhances the rate of evaporation of water droplet on solid substrate [47].

Can the water droplets be excluded from nonpolar nanocavities by applying electric fields? To address this question, in this paper, systematical studies were performed on the electric-field-inducing dewetting of hydrophobic nanocavities between graphene walls exposed in atmosphere by means of molecular dynamics (MD) simulations. Rapid dewetting of water droplet in nanocavities is found to be induced by electric field at ambient temperature. In comparison, dewetting induced by high temperature exhibits low rate even when the temperature reaches to 423 K. Also, the water molecules in vacuum chamber induced by electric field were found to form the longitudinal column array rather than the vapor. Our results demonstrate a possible route to dehydrate the nano-porous material by electric field.

## 2. Materials and Methods

**Figure 1:**
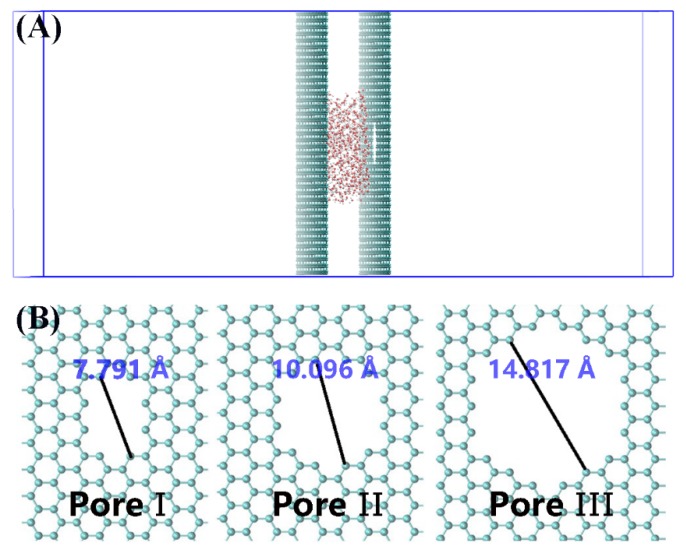
(**A**) Side view of the computational system investigated in this work. (**B**) Typical graphene pores named as Pore-I, Pore-II, and Pore-III by removing 10, 24, and 54 carbon atoms, respectively. Gray spheres represent carbon atoms and red spheres and green spheres represent the oxygen and hydrogen atoms of a water molecule, respectively. The vacancy on graphene represents the nanopore in Figure 1A.

The simulation system in classical MD simulations includes an orthogonal box with dimensions of 96.995 Å × 84.000 Å × 200 Å. 509 water molecules are initially located between two graphene walls along the x-y plane with the separation of 20 Å (as shown in Figure 1A). The systems with different numbers of water molecules and the separation of 40 Å are also examined for testing simulations which give same qualitative results. The periodic boundary conditions are applied in all three dimensions to mimic the micro-sized nanocavity and the vacuum layer along the z direction (~180 Å). Although a perfectly crystalline graphene sheet is impermeable, nanopores of various diameters can be realized in graphene. Here, we design three nanopores by removing a few carbon atoms of one graphene to study the dewetting process of water droplet confined between two graphene walls (Figure 1B). Three nanopores with diameters of 7.791, 10.096, and 14.817 Å are built, named as Pore-I, Pore-II, and Pore-III, respectively. Moreover, the nanopore with the diameter of 5.6 Å is also studied. However, dewetting is not observed under high temperature and electric field. Due to the water droplets can move easily in the nonpolar nanocavities [41,42], the nanopores are induced on the top of water droplet to improve the simulation efficiency (Figure 1A).

Classical MD simulations were performed with the Gromacs 4.5 package [48]. A simple point charge-extended (SPC/E) model was used for water [49]. The carbon atoms are modeled as uncharged Lennard-Jones particles with σ_C_ = 3.55 Å and ε_C_ = 0.29288 kJ/mol. The Lennard-Jones interactions are calculated using Lorentz-Berthelot combination rules. The energy parameter of ε_C-O_ = 0.436 kJ/mol results in the contact angle (~85°) of water on the graphene sheet. In general, 90° has been considered the demarcation between hydrophilic and hydrophobic characters. However, numerous previous studies show that graphene is hydrophobic with a water contact angle in the range of 84–127° and the decrease in ε_C-O_ leads to the increase in contact angle value (i.e., an increase of the hydrophobic character of the graphene surface) [50,51,52,53,54]. Thus, we consider contact angle 85° closer to 90° to be weak hydrophobic in this work. We also test other parameters with bigger contact angle (hydrophobic), and same qualitative results are obtained. All the simulations are performed in NVT ensemble using the Nose-Hoover thermostat. A cutoff value of 12 Å is used for the van der Waals interactions, and the long range electrostatic forces are calculated using the particle-mesh Ewald method (PME). A time step of 2 fs is used to integrate the equations of motion by leapfrog algorithm. Each system was equilibrated for 10 to 30 ns depending on the dry process. During the simulations, carbon atoms were frozen to their lattice position to prevent out-of-plane displacement. 

## 3. Results and Discussion

**Figure 2:**
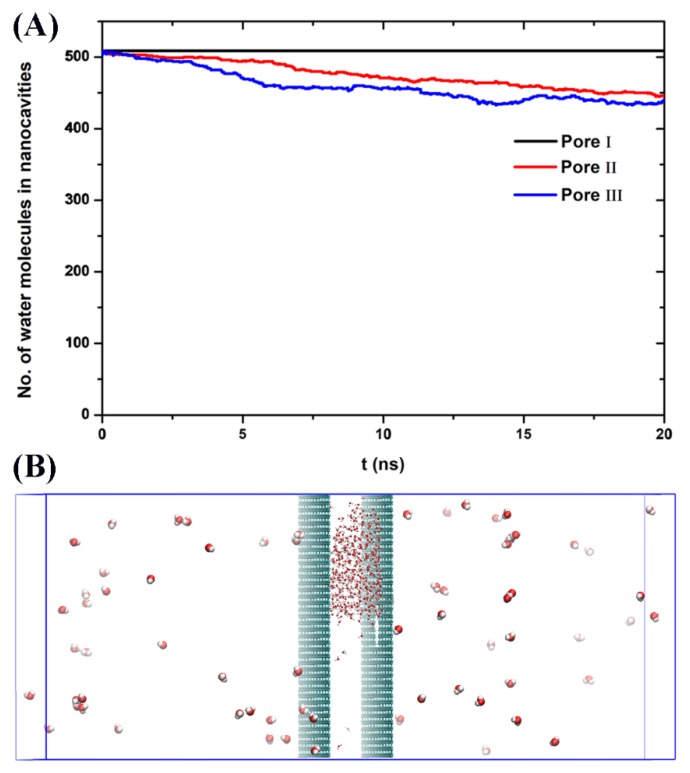
(**A**) The number of water molecules between the graphene walls at 423 K during the simulation for Pore-I, Pore-II, and Pore-III, respectively. (**B**) The side view snapshot of the simulation system for the case of Pore-III at 423 K. Gray spheres represent carbon atoms and red spheres and green spheres represent the oxygen and hydrogen atoms of a water molecule, respectively. The big spheres represent the water in vacuum chamber. The vacancy on graphene represents the nanopore in Figure 2B.

MD simulations were first carried out to study the thermal dewetting of the nanocavities by increasing the system temperature. Unlike boiling (water boiling point of 598 K for SPC/E model), the dewetting rate of nanocavity in this work is defined as the number of water molecules which enter into the vacuum chamber through the nanopores on graphene at a certain period of time. The kinetic energy of water molecule cannot offset the intermolecular interactions between water–water and water–graphene walls at ambient temperature in the absence of electric fields. Thus, the dewetting cannot occur. With the increase of temperature, the kinetic energy increases. When the temperature reaches to 423 K, the number of water molecules between the graphene walls is also kept constant in case of Pore-I during the simulation of 20 ns (Figure 2A), indicating that there is no water molecule passing through the Pore-I into atmosphere (vacuum chamber). That is, dewetting cannot be happened for Pore-I. On the other hand, for Pore-II and Pore-III with the diameters of 10.096 and 14.817 Å, depletion of water molecules confined between the graphene walls can be observed at 423 K (Figure 2A). However, almost all the water molecules are still in the nanocavities (only tens of water molecules evaporated in vacuum chamber during 20 ns). That is, there is a very low rate for thermal dewetting of water in nanocavity though the nanopores. Also, interestingly, it is found that the water molecules in nanocavity form a droplet by interacting with each other, while the water monomers or small clusters are observed in vacuum chamber at 423 K (Figure 2B). 

**Figure 3:**
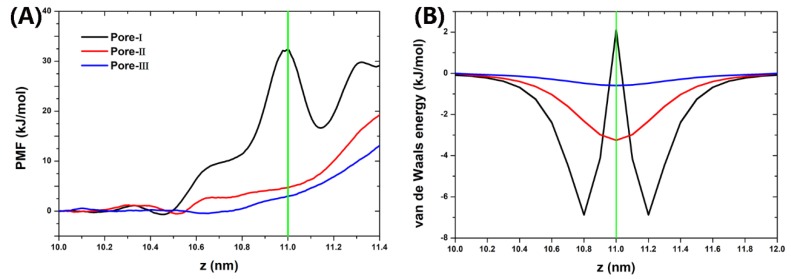
Potential of mean force (PMF) profiles of water passing through the nanopores of graphene along the *z*-axis (**A**). The van de Waals energy between the water and the graphene nanopore (**B**). The green vertical lines represent the position of graphene walls. For PMF profiles, the z < 11.0 nm region represents nanocavities containing water droplet, and the z > 11.0 region represents the vacuum chamber.

To understand the low dewetting rate induced by high temperature, we first calculate the potential of mean force (PMF) of water along the *z*-axis (see detail in Supporting Information). Because the water rarely enters the vacuum chamber through the nanopores of graphene under equilibrium condition in absence of electric field at 300 K, a water molecule was positioned at various locations along the *z*-axis and umbrella sampling was used. As shown in Figure 3A, the center of nanocavity (z = 10.0 nm) was taken as the reference position where the PMF is zero and z = 11.0 nm represents the position of graphene nanopore. The energy barrier (defined as the difference in the PMF between the water in nanodrop and in vacuum chamber) is about 32.5 kJ/mol for Pore-I, 19.5 kJ/mol for Pore-II, and 12.8 kJ/mol for Pore-III. Also, a valley of PMF for Pore-I at z = 11.2 nm corresponds to the strong water-graphene interaction (Figure 3B). Also, we found that the water molecule positioned in the vicinity the nanopore is interacted with the water droplet in nanocavity by hydrogen bonding network (Figure S1). These results indicate that the water droplet in nanocavity is stable.

The electric field can elongate the water clusters or water droplets along the field direction [55,56,57]. Thus, we next study the effect of electric field on the dewetting of nanoconfined water droplet at ambient temperature. The uniform electric fields of 1.5–3.0 V/nm are applied perpendicularly to the graphene walls. This range of the field is at least 1 × 10^3^-times stronger than the external fields applied in the electrofreezing experiments (~10^−3^ V/nm) [33]. We noted that the field strength in our simulation is of the same order as that theoretically predicted for alignment of water dipoles and crystallization into polar ice (>1 V/nm) [30]. Also, the fields are below the critical fields of dissociation of water [58]. And these values are representative of field strength in various nanoconfined environments [32,34,35], as they are comparable to those experienced by water within the crevices of polar crystals, or within molecular distances from charged or polar surfaces of proteins [59,60,61].

**Figure 4:**
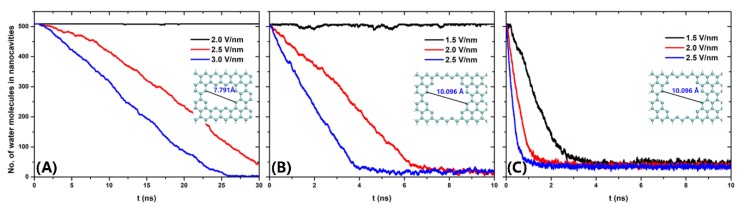
The numbers of water molecules between the graphene walls during the simulations for the cases of Pore-I (**A**), Pore-II (**B**), and Pore-III (**C**), respectively.

In Figure 4, we plot the number of water molecules in nanocavities as the function of simulation time under different electric fields for Pore-I, Pore-II, and Pore-III, respectively. In the case of the smallest pore (Pore-I), few water molecules can be pushed out from the nanocavity at *E* = 2.0 V/nm during our whole simulation of 30 ns (in Figure 4A), similar to the result in the absence of electric fields (Figure 2A). It is remarkable to find that the dewetting transition occurs when the electric field strength reaches to 2.5 V/nm. In the case of *E* = 3.0 V/nm, we observed that the number of water molecules in nanocavity decreased sharply from 509 to 2 at about 25 ns. After 25 ns, almost all the water molecules are removed from the nanocavity into vacuum chamber. Much more rapid dewetting transitions are observed for Pore-II and Pore-III induced by electric fields. Note that the rate under *E* = 2.5 V/nm is about 15 ns^−1^ for Pore-I, 120 ns^−1^ for Pore-II, and 400 ns^−1^ for Pore-III. Also, we importantly found that the critical strength of electric field (*E_C_*) for drying transition decreases with the increase of nanopore diameter (i.e., 2.0 < *E_C_* < 2.5 V/nm for Pore I, 1.5 < *E_C_* < 2.0 V/nm for Pore II, and *E_C_* < 1.5 V/nm for Pore III, respectively). Moreover, the number of residual water molecules in nanocavity for Pore-I case is about 2, while those for Pore-II and Pore-III are about 15 and 35, respectively. The increase of residual water molecules for larger pores can be understood from the water column structures induced by electric field. We also studied the systems for other nanopores with different diameters. And no qualitative difference was observed (Figure S2).

**Figure 5:**
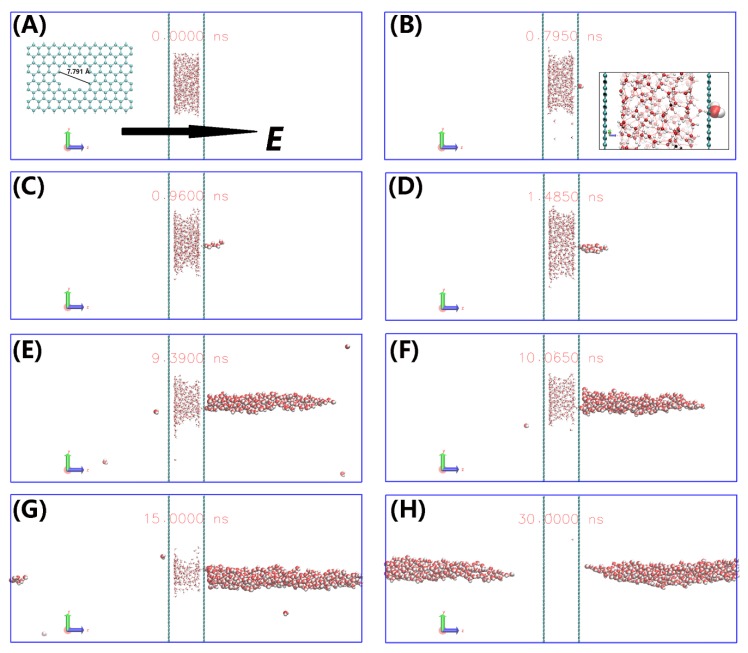
The dewetting process for the case of Pore-I under *E* = 3.0 V/nm and 300 K at 0 ns (**A**), 0.79 ns (**B**), 0.96 ns (**C**), 1.485 ns (**D**), 9.39 ns (**E**), 10.065 ns (**F**), 15.0 ns (**G**) and 30.0 ns (**H**), respectively. Gray spheres represent carbon atoms and red spheres and green spheres represent the oxygen and hydrogen atoms of a water molecule, respectively. The big spheres represent the water in a vacuum chamber.

To gain more insight into the electric field-induced dewetting, the snapshots for Pore I at 3.0 V/nm are presented in Figure 5. The water droplet is confined between two graphene walls in absence of electric field (Figure 5A). When the electric field *E* = 3.0 V/nm is applied, water molecules are removed rapidly though the Pore-I into the vacuum chamber. The water molecules form a single chain when only few water molecules enter vacuum chamber (see Figure 5C). The water chain is consistent with the water cluster structures induced by electric field [55,56]. With the number of water molecules in vacuum chamber increasing, the chain transforms into a longitudinal water column (Figure 5D–H), similar with the structure that water droplet elongated under electric field [47,57]. When almost all the water molecules enter vacuum chamber, the water column separates from the graphene wall (Figure 5H). The threshold field value of 1.5 V/nm for the formation of water column in case of Pore-III (weak van der Waals interaction between water and graphene wall) agrees with the value of 1.2 V/nm for water droplet on the Pt surface [47]. And it requires a threshold field value of >1.5 V/nm for Pore-I and Pore-II duo to the strong van der Waals interaction between water and graphene wall.

We can also find that a chain of water molecules is linked to the end of the column. The residue water in nanocavities is the end of the water column (Figure S3). For Pore-I with small diameter, the tail end is a water chain. Whereas, for larger pores, the tail end is the water column. Therefore, the residual water molecules increase for larger pores. Moreover, there are some water monomers observed in vacuum chamber, although they finally adsorbed in the water column due to the space limitation of the vacuum chamber in simulation. Also, importantly, we can find that the water molecule at the nanopore is interacted with the water both in nanocavity and in vacuum chamber by hydrogen bonding network (Figure S4). We note that the electric field does not produce significant changes in the number of hydrogen-bonds but induce the formation of water column. In fact, the water molecules both in nanocavities and vacuum chamber comply with the ice rules (i.e., every water molecule participates in four hydrogen bonds). However, when the water in the vicinity of nanopore or at the interface of nanodrop, the number of hydrogen-bonds decreases. And the hydrogen-bonds are reorganized to orient the water dipoles more favorably with the electric field (Figure S4).

**Figure 6:**
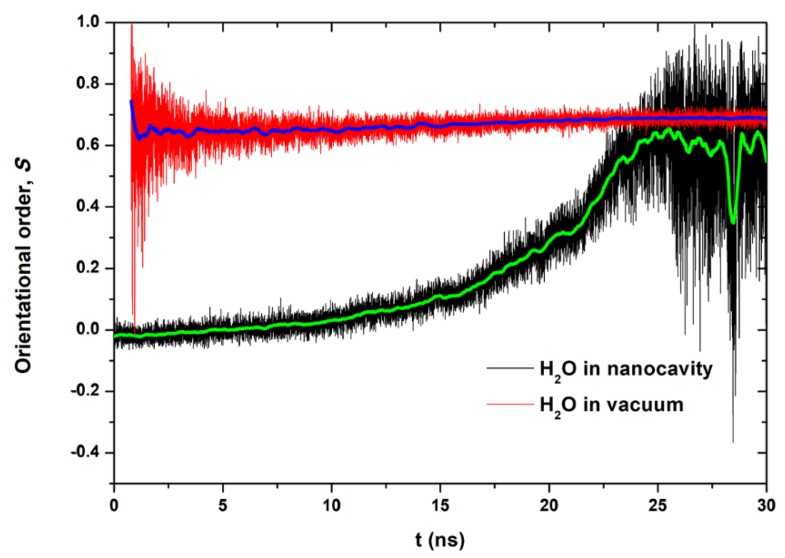
Time dependence of orientational order (*S)* of water molecules in nanocavity and in a vacuum chamber for the case of Pore-I under *E* = 3.0 V/nm.

In order to understand the polarization of water molecules induced by electric field, we investigated the orientational order parameter (*S*), defined as:

(1) $$S=\frac{1}{2}〈(3\cos^{2}\left(\theta \right)-1)〉$$

where *θ* is the angle between the direction of electric field and the dipole moment vector of water molecules. *S* equals 1 and −0.5 for the dipoles aligned with and perpendicular to the electric field, respectively, whereas *S* approaches 0 for the random system. As shown in Figure 6, the water molecules in nanocavity are in a disordered state in the initial stage (*S*~0). As the simulation goes on, the water molecules in nanocavity orientate their dipoles in accordance with the direction of the electric field. On the other hand, the water molecules in a vacuum chamber are always aligned with the electric field because the water molecules have been polarized when they pass though the nanopores. We noted that the orientational order parameter saturates at ~0.68. The reason is that water molecules cannot be completely polarized at *E* = 3 V/nm. A much larger electric field is needed to make the orientational parameter *S* closer to 1. The dramatic fluctuations of order parameter suggest that there are few water molecules in vacuum at initial stage and in nanocavity at end stage. In addition, water molecules confined in nanocavities tend to arrange themselves in layers parallel to the graphene walls, as shown in Figure S5. The layered structures do not affect the formation of a water column induced by an electric field, although it has a bit of influence on the orientation of the interfacial water molecules due to the water–graphene interaction.

**Figure 7:**
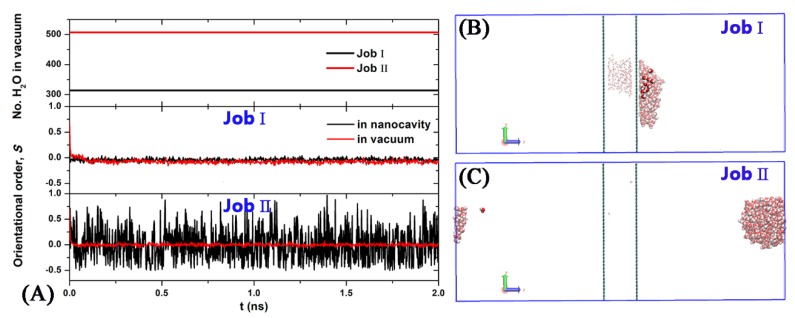
Time dependence of the number of water molecules in vacuum chamber and orientational order (*S*) of water molecules for Job I and Job II (**A**). The side view snapshot of the simulation system at 2 ns for Job I (**B**) and for Job II (**C**).

As described above, an electric field can induce field-alignment of water dipoles and elongate the water droplets along the field direction, which lead to the increase of the dewetting rate. Moreover, the water molecules in a vacuum chamber induced by an electric field form the column rather than the vapor induced by high temperature. One may question what happens if the electric field is suddenly turned off? To address this question, we performed two additional simulations in absence of electric field at *T* = 300 K. The initial configurations are chosen to be the structures obtained at 15 and 30 ns in the case of the Pore-I under *E* = 3.0 V/nm (Figure 5G,H). For the former (Job I), both the nanocavity and vacuum layer contain many water molecules, whereas almost water molecules stayed in the vacuum layer for the latter (Job II). It is shown as Figure 7A that the numbers of water molecules in the vacuum are about constant. That is, there are few molecules passing though the nanopore. Also, the orientational order *S* approaches rapidly to 0, indicating that the water molecules in nanocavity and in vacuum chamber are in the disordered state when the electric field is turned off. The snapshots of the final configurations are shown in Figure 7B,C. In the case of Job I, the water column in vacuum chamber linked with the water droplet in nanocavity transforms into a water droplet which is adsorbed on the graphene, while in case of Job II the water column separated from the graphene wall transforms into a water droplet with spherical shape in vacuum chamber. 

## 4. Conclusions

In this work, we employed molecular dynamic simulations to study the dewetting of nanocavities induced by electric field and by high temperature. We found that the water in nanocavities can rapidly pass through the nanopores on graphene and move into the vacuum chamber induced by electric field at ambient temperature. However, in the absence of an electric field, a low rate of dewetting is found even when the temperature reaches to 423 K due to the strong interaction of the hydrogen-bonding network of the water droplet in the nanocavities. The rate of E-field-induced drying increases with increasing electric field. Moreover, the water molecules in the vacuum chamber induced by an electric field form the column rather than the vapor. If the electric field is turned off, the water column transforms into a water droplet which is still in the vacuum chamber. As a result, the electric field may be a useful approach for the dehydration of nanoporous materials.

## Supplementary Materials

The following are available online at https://www.mdpi.com/2079-4991/10/4/736/s1. Figure S1: The side view of the snapshot for a water molecule positioned at the nanopore. Figure S2: The side views of the snapshot for Pore-IV under E = 3V/nm at t = 0 ns (A) and t = 100 ns (B). The numbers of water molecules between the graphene walls during the simulation (C). Time dependence of orientational order (S) of water molecules in nanocavity and in vacuum chamber (D), Figure S3: The snapshots for the cases of Pore-I (A), Pore-II (B), and Pore-III (C) under electric field. Figure S4: The hydrogen bonding networks of the system evolution for Pore-I during the simulation under *E* = 3 V/nm. Figure S5: The density profiles of water molecules along the *z*-axis.
