# Supplementary material for: Electric Field Induced Dewetting of Hydrophobic Nanocavities at Ambient Temperature
Source: Nanomaterials (Basel). 2020 Apr 12;10(4):736. doi: 10.3390/nano10040736 (PMC7221969; doi:10.3390/nano10040736)
Supplement: Supplementary file 1 [file nanomaterials-10-00736-s001.pdf]

nanomaterials

Supplementary Materials

Electric Field Induced Dewetting of Hydrophobic Nanocavities at Ambient Temperature

Chenchao Li, Dongdong Lin *, and Wenhui Zhao *

School of Physical Science and Technology, Ningbo University, Ningbo 315211, China; 1711071045@nbu.edu.cn

* Correspondence: lindongdong@nbu.edu.cn (D.L.); zhaowenhui@nbu.edu.cn (W.Z.)

1. Potential of Mean Force (PMF) Calculation

The potential of mean force (PMF) profile for the water molecule along the z-axis is calculated by using the umbrella-sampling algorithm. The force constant adopted in the harmonic biasing potential is 2000 kJ $mol^{-1}nm^{-2}$. The target positions vary from 10.0 nm to 11.4 nm at increments of 0.1 nm (the coordinate of the graphene nanopore is z = 11.0 nm). A total of 15 simulations yield overlapping windows of density probability. Each window is sampled for 6 ns and the last 2 ns of data are analyzed using the weighted histogram analysis method (WHAM).

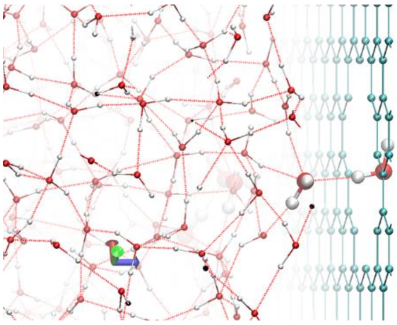

Figure S1. The side view of the snapshot for a water molecule positioned at the nanopore in absence of electric field at 300 K. The red lines represent the hydrogen bonds.

2. The simulation for the system Pore-IV

We also studied the other system including 1410 water molecules confined the graphene walls (Figure S2A). The nanopore on the graphene is named as Pore-IV by removing 16 carbon atoms. The box sizes are 4.92 nm × 5.112 nm × 20 nm. The simulation is 100 ns under 3 V/nm at 300 K, which is long enough. Figure S2 shows that the water molecules in nanocavity are almost removed into vacuum chamber. And electric field-induced water column formation is also observed. Moreover, we found that the orientational order parameter of water in nanocavity saturates at ~0.62, slightly lower than that in vacuum chamber.

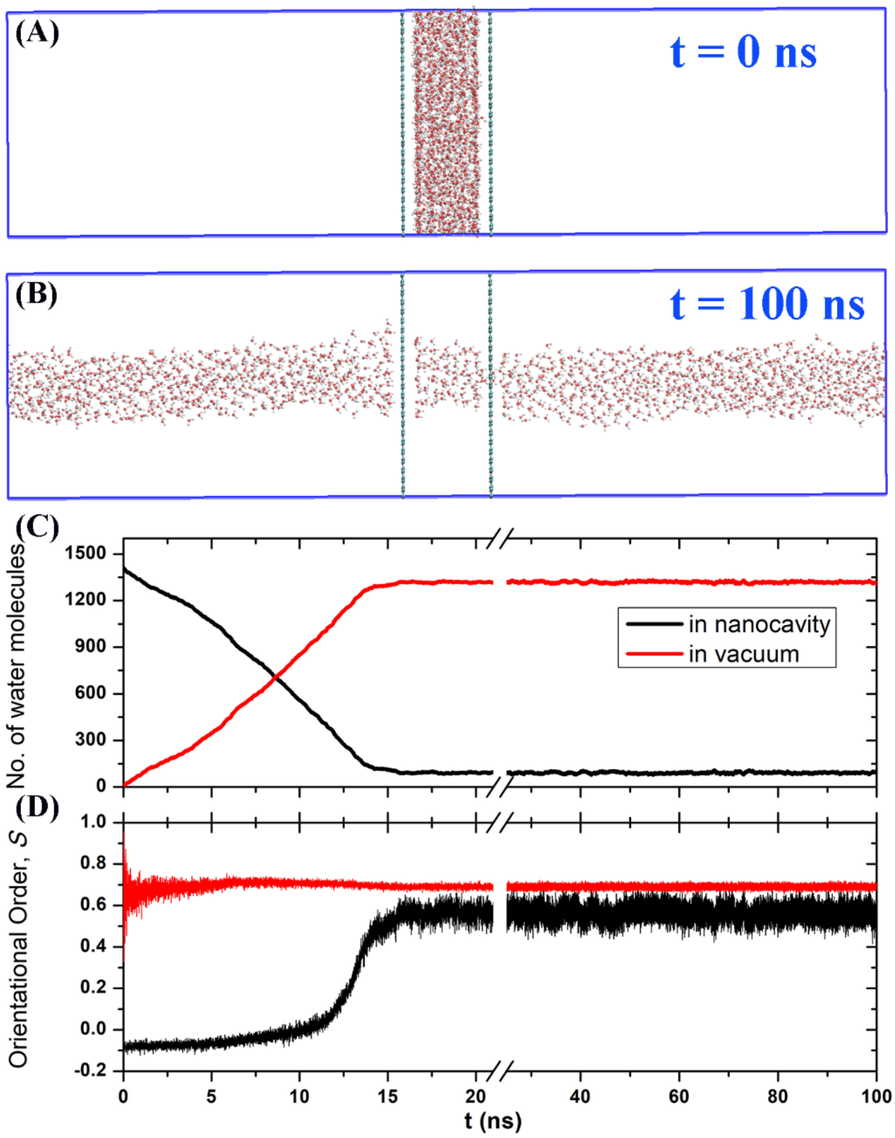

Figure S2. The side views of the snapshot for Pore-IV under E = 3V /nm at t =0 ns (A) and t =100 ns (B). The numbers of water molecules between the graphene walls during the simulation (C). Time dependence of orientational order (S) of water molecules in nanocavity and in vacuum chamber (D).

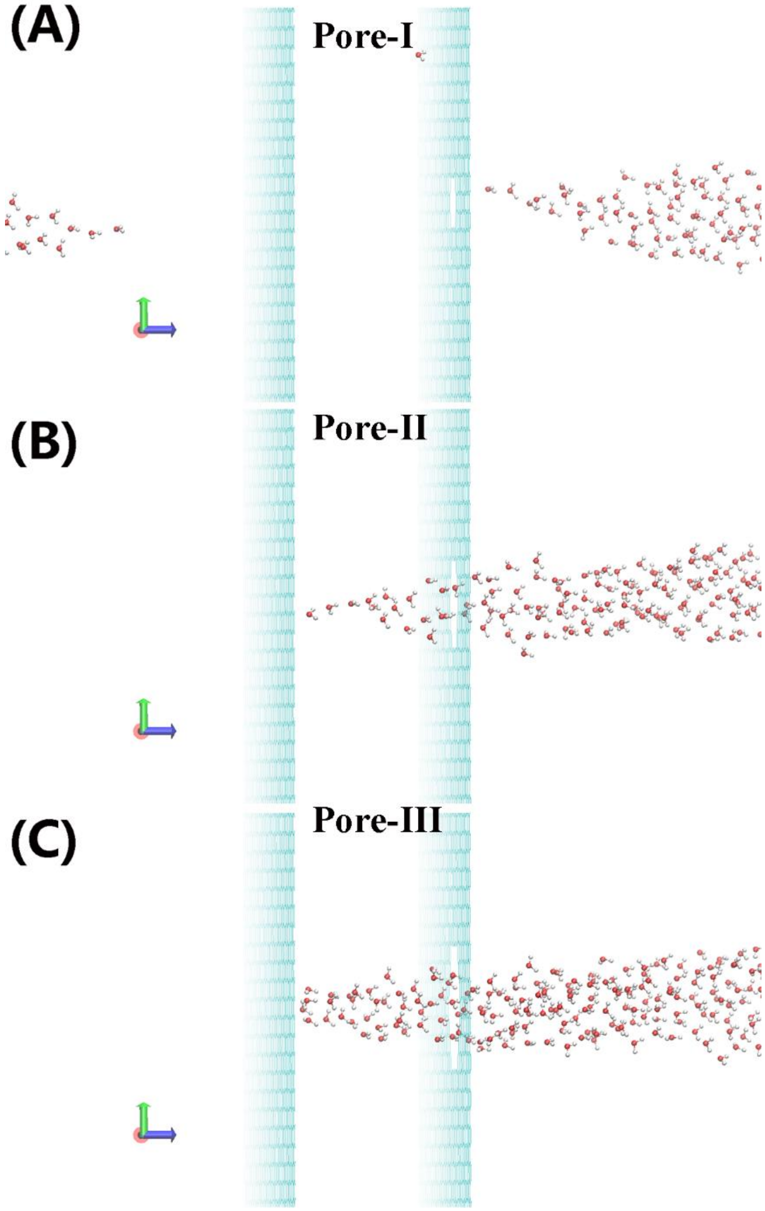

Figure S3. The snapshots for the cases of Pore-I (A), Pore-II (B), and Pore-III (C) under electric field at ambient temperature.

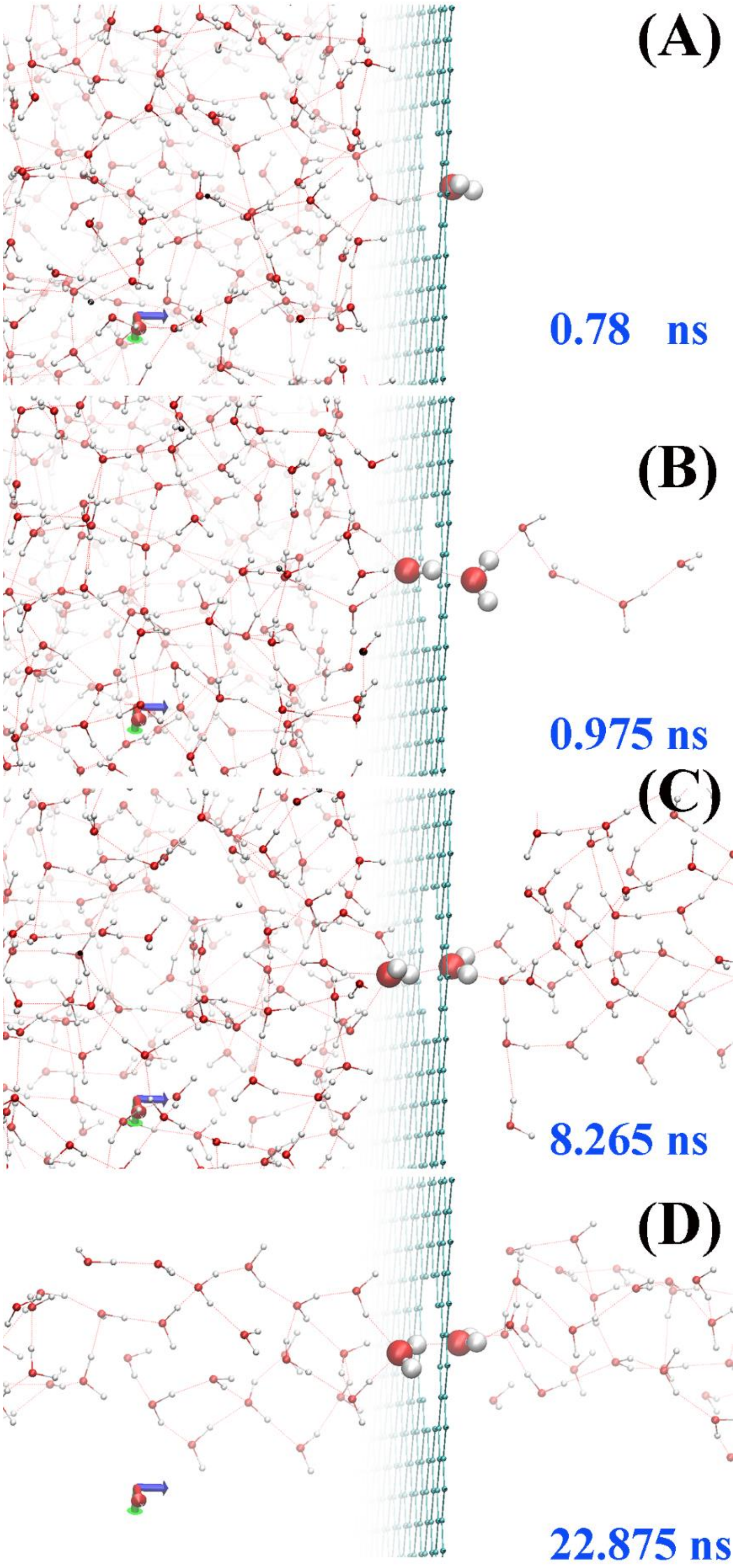

Figure S4. The hydrogen bonding networks of the system evolution for Pore-I during the simulation under E = 3 V/nm. The red lines represent the hydrogen bonds.

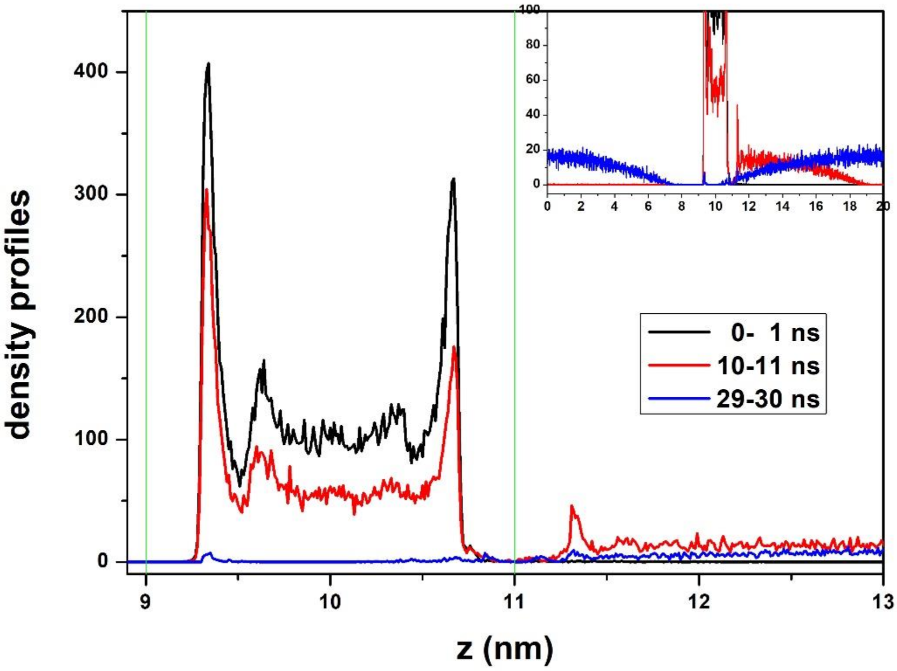

Figure S5. The density profiles of water molecules along the z-axis. The green vertical line represents the position of graphene wall.
